# Perceptions of adolescent pregnancy in the rural context and the Colombian armed conflict: a qualitative approach based on social determination of health

**DOI:** 10.1186/s12939-021-01568-2

**Published:** 2021-10-20

**Authors:** Herlan Palacios-Perdomo, Naydú Acosta-Ramírez

**Affiliations:** 1 VallenPaz Corporation, Cali, Colombia; 2 Universidad Santiago de Cali, Cali, Colombia

**Keywords:** Qualitative research, Rurality, Sexual behavior, Adolescent pregnancy, Health inequities

## Abstract

**Background:**

Adolescent pregnancy in rural areas is a persistent health problem that has still not properly been understood. Studies with qualitative perspectives that address this phenomenon as a complex social process, which involves the recognition of the voices of the actors involved and the analysis of the specific context in which it takes place, are limited.

**Objective:**

This research explored the perceptions of young people and other social actors (municipalities of Palmira and El Cerrito in Colombia) of the social forces and dimensions of the social determination of adolescent pregnancy in the Amaime river basin. These geographic areas have been scenes of armed violence with various groups in combat within the context of a long-standing political conflict in Colombia. After the 2016 Havana agreements were signed, peacebuilding has been underway in its territories.

**Methods:**

A qualitative study that implemented focus groups and semi-structured interviews was conducted. The theoretical approach of social determination of health proposed by Breilh was used to study the social process entailed in adolescent pregnancy. Perceptions about social conditions, specific ways of life, and lifestyles were addressed. Galtung and Fischer’s theoretical approach on violence and peacebuilding was also incorporated to enrich the understanding of the Colombian context. The analysis was conducted with approaches from phenomenology.

**Results:**

Living conditions with strong social stigma and demand for social, political, and cultural opportunities were found. Regarding ways of life, little communication and information about sex education was perceived. As for lifestyles, there are youthful behaviors infused by sociocultural traditions that affect life projects and sexual behavior. Gender relations are precarious, and there are various types of violence that limit effective peacebuilding.

**Conclusions:**

This study contributes to a priority issue in sexual and reproductive health, with an approach that generates analytical elements to comprehensively expand the social and health interventions required.

**Supplementary Information:**

The online version contains supplementary material available at 10.1186/s12939-021-01568-2.

## Background

In Colombia, one out of every five women under the age of 19 has been through a pregnancy. Therefore, comparing world report findings that show that 10% of the young women in that age group has experienced childbirth [1], Colombia is considered a country with a high rate of adolescent pregnancy [2]. According to the National Administrative Department of Statistics of Colombia, in 2019, 4713 children were born to mothers between the ages of 10 and 14 years, and 116,609 to mothers between the ages of 15 and 19 years [3]. Adolescent pregnancy in rural areas represents 24% of all childbirths, and 15.1% occur in urban areas [3].

The Colombian municipalities of Palmira, where 3562 adolescent pregnancies were recorded in 2016, and El Cerrito, which recorded 543 pregnancies [4], stand out. These municipalities are part of the Amaime river basin, featuring an extensive rural area. These geographical areas used to be scenes of armed violence within the framework of a long-standing political conflict in the country. This took place with high intensity in the time period preceding the Havana Peace Agreement (signed on September 26, 2016) and is currently seeking peacebuilding in the nation [5]. The population experienced armed violence due to the confrontation of different groups in combat, such as the guerrillas with a political leaning toward the Marxist–Leninist left called the Revolutionary Armed Forces of Colombia—People’s Army (FARC-EP), the far-right political armed forces called the United Self-Defense Forces of Colombia (AUC), and the military forces of the national government [5].

In general, the phenomenon of adolescent pregnancy is relevant due to its social and health implications, as it is concerned with school dropouts and increased maternal mortality [2]. As a result, since the 1970s, adolescent pregnancy has been considered a priority issue in public health, requiring comprehensive approaches that incorporate the analysis of the specific context and its reinterpretation as a complex social process. This means recognizing that it is a process with multiple interrelated factors involved and that this process takes place in a social environment where various institutional and individual actors are interconnected—in a historical moment and in a particular space [6, 7].

Studies aimed at addressing the phenomenon of adolescent pregnancy as a social process in rural areas are limited, and those focused on scenarios of violent armed conflict and transition toward peacebuilding are even more scarce. In Colombia, some studies indicate that women in these contexts were subjected to sexual violence that triggered unplanned pregnancies [8].

In Latin America, some studies have shown that the phenomenon of adolescent pregnancy is related to the contexts of poverty, low education levels, and social inequalities [6]. Research has shown that there are both sociocultural components and individual emotional elements, which are present in both rural areas and urban settings, where adolescence is constructed as a social and historical process between traditional and modern elements [2, 6, 9, 10].

Despite recognizing the social relevance of equity in developing human capital and increased social expectations with diversified life projects for women in urban groups with a medium and high socioeconomic level, in the rural context, social inequities are present because a reproductive role assigned to women prevails at an early age, strengthened by processes of primary (home) and secondary (other spaces) socialization [11].

Evidence has shown that early fatherhood and motherhood are not independent of the sociocultural and economic contexts of societies and the population subgroups that comprise them [6–11]. Becoming a parent is mediated by the experiences that each person has, where constructing the meaning of motherhood and fatherhood is determined by patterns of the ideological, cultural, and religious order of the social context, which impact their interactions in daily life and its discourse [6, 9].

Qualitative methods are appropriate to analyze these complex phenomena by addressing the knowledge of representations, meanings, and perceptions from the perspective of individuals as a field of study. This is highlighted in various studies aimed at young people and the area of sexual and reproductive health [6, 8, 9, 12, 13]. Studies on perceptions seek to obtain information about the values, traditions, experiences, prejudices, ways of thinking, and knowledge that adolescents have in their family contexts and in their specific socioeconomic and cultural environment [6, 13, 14].

The research question was “What are the perceptions of the experiences of young people and social actors in the Amaime river basin regarding the social forces and dimensions of social determination involved in adolescent pregnancy in scenarios of violence and peacebuilding?”

The need to understand the phenomenon of adolescent pregnancy is recognized in this study, incorporating comprehensive perspectives based on the perceived demands and characteristics of the specific social contexts in these population groups [13]. The purpose is to obtain analytical elements that lead to rethinking proposals aimed at transforming and improving actions to promote and prevent young people’s sexual health.

In this sense, the limitation of both these approaches focused on considering isolates individuals and outside a complex social context is also recognized, as well as the limitations of subsequent interventions based on promoting the use of contraceptives as central actions, and which have traditionally been used to face the phenomenon of adolescent pregnancy without considering other collective health actions derived from alternative analyses with holistic perspectives, such as those from the social determination approach [2, 6].

This study aims to contribute to a broader vision of the phenomenon of adolescent pregnancy by transcending the theoretical approaches of studies on “health determinants,” which favor perspectives with traditional epidemiological foundations [15]. In contrast, the theoretical foundations of the social determination approach are used in this study based on Breilh’s proposal [16], as these are used to address the perceptions of social actors and inquire about the context and social process where problems are produced and reproduced and where social inequities arise [16]. This study frames the exploratory analysis of the adolescent pregnancy issue in the principles and challenges of health promotion in accordance with the provisions of the Ottawa Charter that highlights the relevance of the analysis and intervention of social aspects in the health–disease–care process.

## Methods

### Study context

**Fig. 1:**
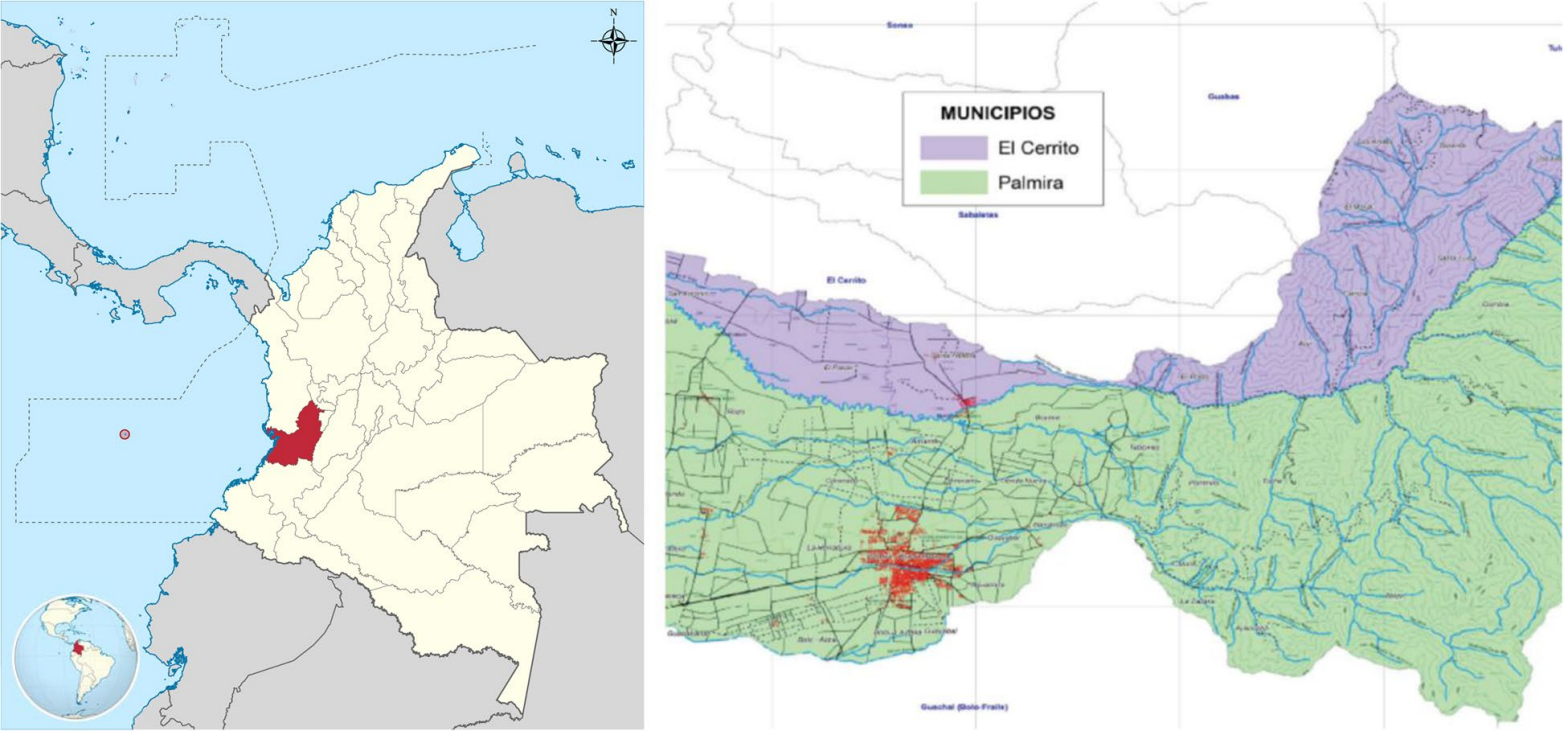
Localization of Amaime river basin (municipalities of Palmira and Cerrito in Colombia). Source: Adapted and edited for our work by the authors. From: www.todacolombia.com and https://www.cvc.gov.co/documentos/planes-yprogramas/planes-de-ordenacion-y-manejo-de-cuencas-hidrografica/amaime

This study was carried out in the Amaime river basin located in the southwestern region of Colombia (between the municipalities of El Cerrito and Palmira), with an area of 104,291 ha (Fig. 1). Colombia’s rural areas experienced differential armed violence as a result of the conflict between opposing political groups [5]. Some populations suffered more than others, and among these are the inhabitants of the Amaime watershed. The Amaime river basin has suffered from the Colombian armed conflict for decades, with an upsurge between 2006 and 2016, the period preceding the peace agreement that was signed during President Juan Manuel Santos’s administration. The conflict is related to territorial and population control in areas considered strategic by the groups in conflict, due to factors that include formal and informal mining, arms trafficking, and drug trafficking routes.

### Theoretical framework

In this study, the analytical elements proposed by Breilh in the social determination approach were adapted [16] in addition to the incorporation and adaptation of Galtung and Fischer’s approaches to violence [17].

The approach to social determination proposed by Jaime Breilh aims to understand health-disease as a dialectical process in which individual health is linked to collective health. As a result, different domains are incorporated as dimensions of analysis, which range from singular elements to general aspects. The subjects coexist with structural social conditions, which are related to the lifestyles of the population groups or subgroups, and finally these factors determine individual lifestyles [16].

Breilh’s approach focuses on the analysis of the forms of social organization and economic and social structures, in which the identity of specific groups is constructed and which give rise in parallel to the worldview and subjectivity of individuals. Together, these elements make up the ecological or eco-systemic space where human beings develop, thus revealing the roots of inequality and inequities in health [16].

As a result, the present study will explore the phenomenon of adolescent pregnancy transcending the traditional epidemiological approach that has been criticized for focusing on the quantitative analysis of isolated variables. It will adapt Breilh’s perspective of social determination [16] to recognize the living conditions, ways of life, and lifestyles of the subjects involved. These dimensions of analysis are constituted in spaces of social reproduction that affect the identity of the community and bond development between them, and where an interaction between visible and invisible forces of power relationships generate healthy or unhealthy processes of individuals and their communities [16].

As the study is carried out in a geographic area that used to be a scene of violence and armed conflict—the Amaime river basin—the perspectives of Galtung and Fischer are also incorporated into the study [17]. These authors argue that in peacebuilding contexts, it is necessary to overcome the negative paradigm of peace focused on quantifying deaths due to violent conflicts and the response by signing agreements to end war, leaving out the analysis of the structural and social justice factors that lead to its origin. They propose the concept of positive peace, which implies the transformation and intervention of conflicts, analyzing the various types of violence and the mechanisms that generate it [17].

**Fig. 2:**
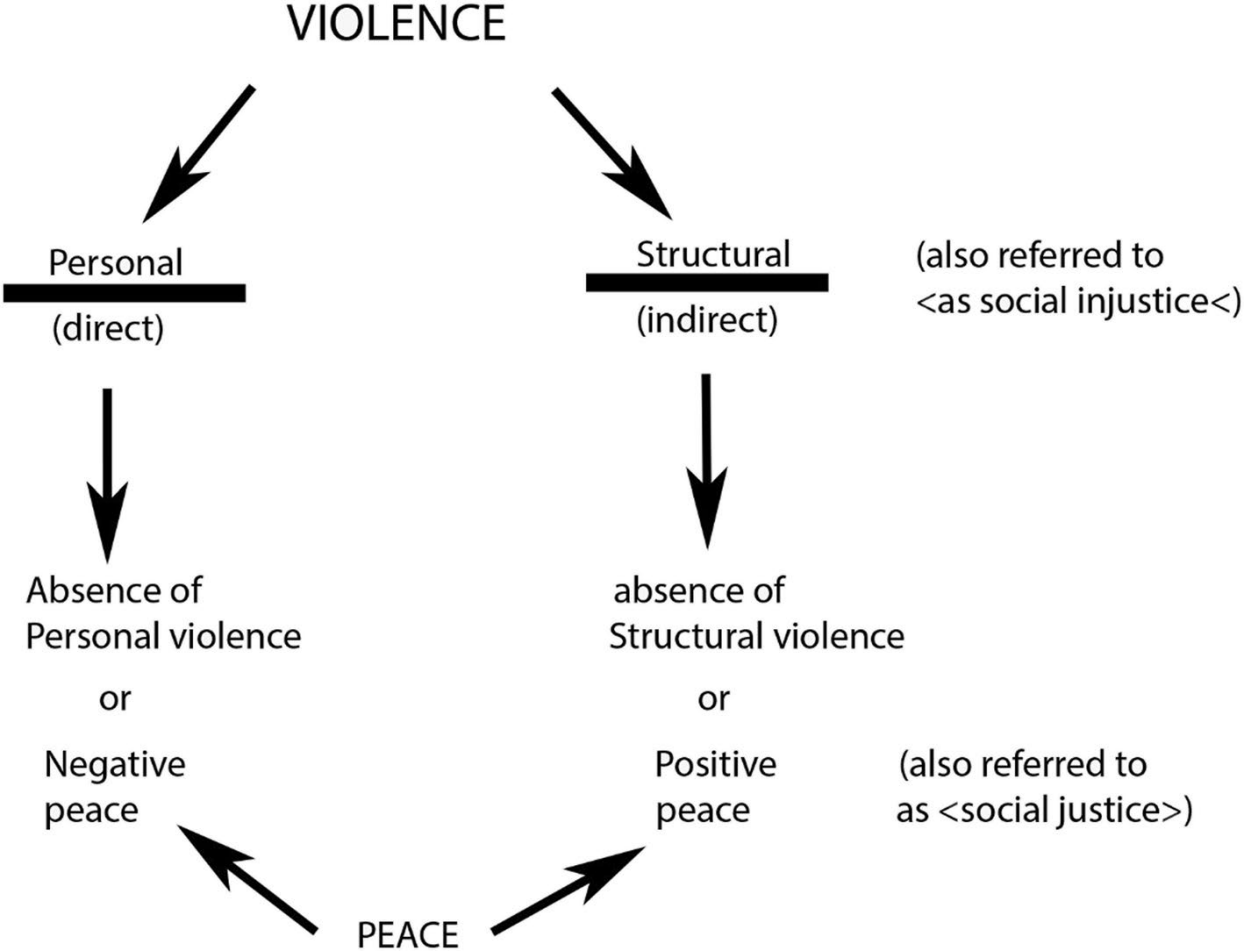
Types of violence and social justice, according to Galtung's proposal [18]. From: Galtung, Johan. "Violence, peace and peace research." Organicom 2018; 15(28): 33-56.

Galtung and Fischer argue that there are direct (visible) violence, structural (invisible) violence, cultural violence, and symbolic violence (Fig. 2). Direct violence is related to the use of weapons or visible mechanisms (for example, a blow with the hand or foot, the use of rifles and other weapons) that cause physical injury to individuals. Meanwhile, structural violence is associated with the society’s basic needs, and when a neglected or an inadequate response is offered, they become generators of poverty and misery for socially relegated population groups [17].

In turn, cultural violence is expressed in various forms of domination, with the contempt or devaluation of knowledge of minority subgroups (for example, ethnic groups) in both academic fields and in law or regulations, and where they are supposed to promote the dignity of all human beings, without discrimination [17]. Symbolic violence is manifested in stereotypes and social stigma and comprises invisible forms based on unified codes of conduct that generate barriers and exclusion for those with ways of thinking and acting different from the predefined parameter [17].

To overcome these violence and the problems related to it, Galtung and Fischer proposed the ideas of social justice, the conflict intervention and transformation, the vindication of human rights, and the peace and healthy coexistence-building approach [17].

### Study design

A qualitative and exploratory study was carried out with an approach using elements of phenomenology [19] to investigate and recognize the perceptions of the social actors involved in adolescent pregnancy in the rural area of the Amaime basin. Social actors are those who have experienced a phenomenon in the midst of their feelings, beliefs, and values and are therefore capable of narrating their experiences from their felt and lived perceptions while simultaneously giving phenomenological meaning to it [19]. As a result, the phenomenology-based approach in the collection and inquiry process leads to the recognition of the interaction between the subjects who participated in the experience of a phenomenon and enables researchers to understand and interpret the phenomenon from the actors’ daily experience [19].

### Ethical considerations

This project was approved by the ethics committee (endorsement document No. 021–18) of the university to which the main researcher was affiliated during postgraduate studies (professional graduate in Political Science, with previous experience in community projects in the study area) and the co-researcher (Doctor of Public Health Sciences) was serving as director of the degree work of the Master of Public Health program. The application of informed consent and assent is highligted, as well as the support provided by a professional in social psychology, to manage crisis situations with the participants, given the complexity of the experiences of adolescent pregnancy.

### Population and sample

The population considered for the study comprised residents in the rural area of the Amaime river basin, using a type of purposeful or intentional sampling, typical of qualitative studies [20] aimed at selecting the social actors that could provide the greatest wealth of information to study the research question. The inclusion criteria were young men or women who have faced parenthood at an early age (between 14 and 18 years).

Considering that other relevant social actors coexist in this study phenomenon, constituting key informants for the study, other population groups included were community leaders, health personnel assigned to those localities, and health authorities in the municipalities from Palmira and El Cerrito. The sampling used was also intentional [20], with the inclusion criteria of residing or working in the areas studied. The exclusion criterion for all population groups was that they did not sign the informed consent form or the assent statement for their participation in the study.

### Recruitment of participants

The process of locating and enrolling the participants for the project was performed by the main researcher by visiting the public health institutions in the geographic area of study, the government education institutions in the area, and two non-formal training centers, where young people and adults from the families in the Amaime basin participate in continuing education processes, organized by an international foundation. Approaching various organizations and social actors enriched the participation of key informants according to the required inclusion criteria.

The number of participants was influenced by applying the saturation strategy [20], which in qualitative studies involves stopping the enrollment process when there is redundant data from participants regarding the study phenomenon.

Two young women who experienced adolescent pregnancy refused to participate in the study due to a lack of time because of their duty in farms. In two other cases, referred by the health institution of the study area, mothers did not allow their pregnant adolescent daughters to participate. Other difficulties and obstacles mentioned by four social actors invited to participate in the research, and who ultimately declined, were limitations in transportation to the education institution where they were to meet, because the study area is a vast, rural, and remote area with a very dispersed resident population. Thus, the results of this study are applicable and limited to specific groups with characteristics similar to the population involved (see Tables 1 and 2), constituting a limitation of this study.

**Table 1:** Characteristics of the participants in the focus groups

Population segment	Municipalities	F: female M: male	Age range (years)
FG1: Social, community or teacher leaders	Palmira	F = 5, M = 3	30–60
	El Cerrito	F = 4, M = 1	30–60
FG2: Young people with adolescent pregnancy	Palmira	F = 2	14 y 18
	El Cerrito	F = 2	14 y 18
FG3: Young people with close experience of adolescent pregnancy	Palmira	M = 4	25–30
	El Cerrito	F = 3, M = 1	25–30

Source: Created by the authors

**Table 2:** Characteristics of the participants in the semi-structured interviews

Population segment	Municipalities	# of interviews	Sex F: female M: male	Age range (years)
Health sector	Palmira	2	F: 2^(b)^	25 y 37
Director or manager^(a)^	El Cerrito	2	M: 1^(a)^	50
Responsible for sexual and reproductive health program^(b)^			M: 1^(b)^	50
Social leaders	Palmira	2	M: 2	35 y 49
	El Cerrito	1	M: 1	25 y 30
Young people pregnant or couple with that event	Palmira	2	F: 1, M: 1	14 a 18 18
	El Cerrito	1	F: 1	
Young people with close experience of teenage pregnancy	Palmira	1	F: 1	19 a 29
	El Cerrito	1	F: 1	19 a 29

Source: Created by the authors

### Data collection and analysis

Focus groups and semi-structured interviews were used to collect information with the support of field notes. In total, 3 focus groups with 25 participants (see Table 1) and 12 semi-structured interviews (see Table 2) were conducted during the months of February and March 2019. The average duration for the focus group was 2 h, while the semi-structured interviews lasted between 45 min and 1.5 h.

For the purposes of this study, seven analytical categories adapted by the authors (see Table 3) were initially developed based on the proposal of the three domains of Breihl’s approach to social determination [16] and Galtung and Fischer’s analytical perspective on violence [17]. These categories were addressed in the research through guides with open-ended guiding questions, developed both for conducting focus groups and semi-structured interviews (see Additional file 1).

**Table 3:** Predefined analytical categories for the study

Domain	Category
General.	Living conditions
Specific	Productive social forces Ways of life Community identity
Singular.	Lifestyles
The scene of violence.	Types of Violence Peacebuilding

Source: Adapted by the authors for this study, based on the proposal by Breilh [16], and Galtung & Fischer [17]

The theoretical saturation technique was used to collect and process information [20], considering the contribution of new data to identify emerging analysis subcategories that need to be analyzed and interpreted differentially. Thus, the emerging subcategories correspond to unexpected elements or information not considered in the initial design, indicated and attributed to the phenomenon studied by participants. The 11 subcategories identified in the data analysis were incorporated into the framework of the social determination domains, which broadened the understanding and interpretation of the phenomenon (see Table 4).

**Table 4:** Emerging subcategories in the study

General domain	Specific domain	Singular domain
**Living conditions category** The local economy in rural areas Education. The political dynamics	**Ways of life category** Adolescent care Motherhood and fatherhood Health and education services Local social forces	**Lifestyle category** Family communication and bond Responsible decision-making Relationships and union as a couple at an early age Virtual social networks

Source: Created by the authors, adapted for this study based on the proposal by Breilh [16], and Galtung & Fischer [17]

The analysis began with the audio transcription of the interview and focus group recordings, using text files created with Microsoft Office Word Professional Plus 2013. The information organization process was completed separately in a matrix for the young actors who experienced adolescent pregnancy and another for the other social actors, using differential alphanumeric codes to preserve participants’ confidentiality. Subsequently, the information was processed and added to the seven predefined categories and the 11 emerging subcategories (using the tool to include comments from Word) according to the perceptions, conditions, circumstances, or situations reported by the various study participants.

During the analysis process, the emergence of repetitive elements in terms of similarities and differences were considered, both in the interviews and the focus groups, with continuous comparison processes, which is a strategy of qualitative methods [20, 21]. The relevant data from this analysis process were extracted to present the results and discussion, applying the analysis strategies and techniques of academic rigor established for qualitative methods [21]. Thus, based on the diversity of information sources (the social actors involved as participants in the study) and the different data collection techniques mentioned, the triangulation strategy was used in the process of analyzing the phenomenon of adolescent pregnancy [22].

Regarding the study’s quality control strategies [21], in addition to a moderator (the main researcher), an observer (a psychology professional) also worked with the focus groups. After the information had been systematized and analyzed, the results were then shared with the study participants at a meeting held for sharing feedback on the research findings, which was then used to identify and incorporate relevant and consensual elements from the perspective of those participants, in order to complement the exploratory analysis of the phenomenon.

## Results

Next, the research results were presented according to the various domains proposed by Jaime Breilh on the theoretical approach to social determination (general, specific, and singular domains) [16], while the findings of the scene of violence are presented in a category considered transversal, as it pervades the social environment where adolescent pregnancy is experienced by the study participants.

### General domain

In the general domain, in terms of the category of living conditions, the subcategory of local economy in rural areas emerges, and the voices of young people and community leaders show that they face difficulties in generating income and obtaining employment. Job opportunities are focused on the agricultural sector, and many young people have higher income expectations than those offered to laborers in planting and processing sugar cane, which is the work usually found in the lowland areas. Further, young people do not want to work in horticulture on the hillsides of the Amaime river basin because of the low income. The participants explained the following:

*“A day’s wage is 35 thousand for onions. [ … ] workers are from Nariño and Cauca—cheaper labor—and that’s why they’re hired.”(Social leader, Interview L3)*

*“Economically, there are shortcomings because [ … ] it’s what they plant and what others pay them for what they sow. They can’t set the price at what they want to be paid.” (Young participant, Interview JE1)*

For the study participants, the opportunities depend on those labor relations, where the social forces of agricultural production are characterized by the concentration of land ownership with the precarious social conditions that are generated, affecting their living conditions with low income for families and limited access to education and health. The subcategory of education emerges, with poor social conditions that are related to early sexual activity and school dropouts of young people.

*“Economically, people in the Amaime river are a bit poor. It’s quite complicated in the field of education because some parts are very far away, and it’s difficult for them to travel. Many of them don’t have the option to live well due to lack of work or lack of opportunities.” (Young participant, Interview JA1)*

*“The living conditions in the communities are not good due to the lack of work, [...] The issue of education has become difficult for me, [...] getting pregnant as a teenager ...” (Young participant, Interview JA1)*

The subcategory of the political issue also arose in the analysis. Young people state that their parents are used in the electoral process and that politicians are not interested in their voice or needs, developing a type of structural violence due to the State’s neglection. Social leaders express social resentment over the State’s lack of presence as an institution to solve socioeconomic deficiencies and as a violent armed actor that participated in the war against the FARC-EP, without making social investments in that geographic area. They also perceived the existence of a high social stigma for having been in the middle of the armed conflict. When asked about armed violence, participants confirmed that demobilization is a factor that makes them feel safer to move freely around the area now. They feel manipulated by the different agents of the armed conflict.

*“It’s no secret … drug control, cattle raiders, and thieves. Now with the peace treaty, it’s good because [ … ] people didn’t go up; they were afraid to go there. The displaced people returned. You can ride on your motorcycle at night and there’s no restriction in the territory that keeps you from walking around. Now, control by the government is needed at the rural level.” (Young participant Fn, focus group 3)*

*“There’s a lack of help with everything, in health care, education, etc. Rural people are unprotected.” (Young participant Jf, focus group 3).*

From the perspective of health representatives, adolescent pregnancy is determined by the aforementioned elements of the rural economy model, a lack of education, and political disappointment, which generate a context of poverty and social resentment.

*“All the young people in the basin [ … ] could be supported [ … ] and be given the education, support, and opportunities that they want. They’re Colombians and are particularly vulnerable, even higher than those in urban areas. The State should immediately implement a public policy for them.” (Health officer, Interview RS2)*

### Specific domain

In this domain, in terms of the lifestyle category, the subcategory of adolescent care emerges from the results of the study. The research participants indicated that the families in the Amaime river basin work in domestic and horticultural jobs in rural areas, while those who live in the plains closest to Palmira and El Cerrito generally work in a variety of positions within those cities. As a result, many young people stay home alone for a long time, except when they are at school. Many claim that there are few cultural events, and that parties and alcohol consumption are the most common options available, and these lead to consequences.

*“Today, adolescent pregnancy is more a reflection of going dancing, because people used to get together with friends to study before; now it’s about going dancing...” (Young participant, Interview JA1)*

In the reports from the participants, like other emerging subcategories, early pregnancy is determined by women being socially assigned the role of motherhood and by the fact that many men do not assume their responsibility regarding fatherhood. A practice is then established in which the difficulties in understanding the relationships of gender equity and equality are evident, where the family forces the woman to assume that responsibility, with few cases in which responsible fatherhood is present. As a result, families taking care of their grandchildren as a result of their daughters’ pregnancies is considered a normal or routine way of life. Adolescent pregnancy involves a high level of responsibility assigned to women, and men are not recognized as having a parallel role and responsibility in this process.

*“I’m speaking from my own experience: I got pregnant when I was 14 years old. My parents supported me, I stopped going to school, and they still continue to help me. I work and live with my parents. I take care of my son; his father didn’t accept responsibility. My son is 10 years old and, little by little, I’m helping him get ahead.” (Young participant AL, focus group 3)*

The study participants indicated that the health care services offered by the public health authorities in El Cerrito and Palmira do not provide young people with adequate coverage, as the area has a very dispersed population in the Amaime river basin. Participants repeatedly mention that there is no access to health information. Therefore, this becomes an additional burden that depends on individual will and is left to the discretion of teachers, within educational institutions. The study revealed that organized community support is also weak due to the dispersion of the rural area that prevents closer relationships among neighbors, which could have been fractured in the armed conflict. According to the young participants, the community ignores how to deal with issues related to adolescent pregnancy. These communities come to play a critical role when pregnancy occurs, developing opinions from their prejudices and beliefs, regarding the issue as a tragedy and recriminating women.

*“ … have it and give it away, that’s what they told me to do with my son. I think they’re ignorant people; they know practically nothing [ … ]. Then if she aborts it, they say that that woman killed him.” (Young participant, Interview JA1)*

As for the characteristics of the area that help understand the community identity, the study participants state that the territory provides them with water and very good soil, as it is a moorland area. However, the geographic isolation, deficiencies in public transportation, political-administrative division between two municipalities (El Cerrito and Palmira), limited access to basic services, and the limited government institution presence converge as factors of social determination of adolescent pregnancy. Perceptions about the community, institutional framework, and family are that they act as negative local social forces by repressing young people’s freedom to be informed and the right to exercise pleasurable sexual activity. As a result, young people seek to educate themselves or get help from other young people who are their social peers.

*“Parents don’t tell you about it [sexual activity]. As parents don’t communicate any of this to us, we look for information elsewhere.” (Young participant, Interview JE2)*

### Singular domain

In the lifestyle category, there is a more unique determinant related to the affective field of adolescents and their self-confidence. Many young people showed a lack of emotional ties and a lack of communication at the family level. Many adolescents remain isolated and do not have spaces and mechanisms to participate in sexual education issues with their families or teachers.

*“ … we leave school, we come home, and often, there’s no older person. Some stay with older siblings, but it’s not the same as having a parent with you to talk to you. That’s what happens with us—most of the time we stay alone.” (Young participant, Focus group 2)*

Communication problems do not allow for the proper processing of reflective self-determination in young people, so they can deal with circumstances of sexual activity and the possibility of adolescent pregnancy by making responsible choices. The participants mentioned that young people are not aware of the risks of pregnancy, and both men and women agree that it is the man who exerts the most pressure to have sex.

*“They’re very open about sexual health; it’s happened that we’ve gone out and the first time they see each other, after one kiss, they’re already dating and everything happens. [ … ] some are very cautious, [ … ] they’re responsible, but others aren’t.” (Young participant, Interview JA1)*

*"We’ve been working with young people to raise awareness about body care and responsibility. Many of the pregnant girls know about the methods; they easily change them for fear that parents know they’re planning. There is a greater understanding of natural methods like the rhythm method. Young people know that planning methods exist, but they haven’t been fully aware of their proper use.” (Health officer, Interview RS1)*

Many young people visualize sexual relationships and their life project as a couple from an early age. Thus, many reports showed that adolescent pregnancy is culturally determined by a rural tradition of dropping out of school and finding a partner at an early age, when women postpone their life plans due to an ideal image of life as a wife. When women become pregnant, they build the bond between mother and child, minimizing the role of fatherhood amid sadness, fear, and the challenge of giving birth while encountering various difficulties.

*“I think you’re perceived as strange, as she got pregnant, I don’t know, that she threw her life away, and who’s the father, [ … ]. At first, I got depressed.” (Young, participant JA3, focus group 2)*

*“If a girl gets pregnant, her life is over; if she wanted to go to school, she can’t do that now, and if she has a child [ … ], she can’t do anything she wants.” (Young, participant JA1, Interview)*

Regarding other aspects of lifestyles, parents and leaders participating in the study point out their concern about how long young people remain connected to virtual social networks (through cell phones) and their influence on the change of attitudes and standardized behaviors that determine how young people act. Older individuals are unable to understand adolescents’ ways of communicating through these codes. Adults mention that young people do not pay attention to adults’ recommendations. They are not interested, and it annoys them. Adolescents are perceived as apathetic and sometimes, they are seen as rude toward adult intervention, especially those who live in the plains of the Amaime river basin.

*“Values and norms have been lost; there’s a lack of responsibility. They spend a lot of time online, and they get angry. They don’t answer when asked something [...] They share music, videos, and pornography. All this has influenced haircuts, manners of speaking, etc.” (Focus group with leaders)*

Older people believe school is the place where they socialize and where their identity is built. Teachers conclude that the health care sector has left the issue of sexual education to schools alone. As a result, the lifestyle that young people are adopting is one of poor communication with their family, broken trust, and involves a loss of the generational bond.

### The scene of violence and peacebuilding

It is easier for young people to talk about peace than it is for adults. The actors’ perceptions show fears and memories of the armed struggle for control of the area. While there is a potential decrease in anxiety about living with armed actors, there are still fears derived from the war, with expressions of sadness and pain, amid a negative perception about the peace agreement and the actual fulfillment of those agreements.

Analyzing the information collected from the perceptions indicated that people experienced both direct violence and symbolic violence exerted by invisible barriers in times of armed conflict, which limited mobility between the middle rural area and the upper area, and that they made it impossible to communicate with each other, in addition to the presence of invisible borders in the populated areas near Palmira and El Cerrito, which persist as a result of violence and urban crime.

*“Communities feel freer to move around, they can think about other expectations, and develop their life project, [...] young men don’t have the pressure to belong to armed groups, [...] they can freely progress in their rural life” (Social Leader, Interview, L1)*

With horror, participants recalled situations of gender-based violence in the armed conflict. However, they consider these to be isolated cases and, in their perspective, they do not see adolescent pregnancy as a product of this context of armed violence. There is a denial of adolescent pregnancies caused by actors in the armed conflict. The community mentions that women of legal age were those who got pregnant by armed actors; and in this case, those who dare to talk about it are the teachers. The rest of the community leaders did not mention it. Among both men and women, there is a need to develop grieving processes, in which psychosocial care is required to recreate violent situations. The problems of cultural violence in terms of gender violence remain hidden, and there is a lack of awareness and a need to educate people on the subject.

The study participants perceive a social stigma of violence, because it is a rural area with a history of armed conflict. They are critical of the situation, emphasizing the government’s responsibility for the low public investment by the State. However, as actors related to war, the participants of this study have suggestions to build a culture of peace. Despite the demobilization process, there is mistrust in the peace process that generates uncertainty about compliance with the agreements and the subsequent investment that is necessary by the State. The peace process’s contribution to tranquility in the area is recognized, although it is considered an imperfect peace as a stronger presence of government institutions in rural areas is needed. The peace imagined by the key actors participating in this study involves transforming complex conflicts, such as drug trafficking, reincorporating former combatants from the armed political conflict, security for the entire population, and increased opportunities in social services and continuing education in the culture of peace.

## Discussion

Through this qualitative research study, with the theoretical framework of Breilh’s social determination [16] and Galtung and Fischer’s violence framework [17], the perceptions of adolescent pregnancy were studied with various social actors in the Amaime river basin. The study was specifically aimed at exploring the dimensions of social determination, the social forces involved, and the various types of violence that occur in relation to this phenomenon in the context of the end of the armed conflict and transition toward peacebuilding in Colombia.

### General findings

This research identifies that social, economic, and political barriers coexist in a complex historical context; and together, these factors act as social forces in the social determination of the inhabitants’ collective health, with specific incidence in reproducing the phenomenon of adolescent pregnancies. As for the perceptions of the population studied, there is a negative concept of municipal governments and the precariousness of the State in preserving human rights, among which those involved in sexual and reproductive health and peacebuilding stand out. The importance of the general dimensions of the social environment in aspects that include the economic, political, and cultural context are also studied by Mora Cancino et al. [2]

From the accounts of adolescent pregnancy in this study, it is clear that this phenomenon is part of rural daily life and is not recognized as an intervening problem. From Stern’s view [23], the problem lies in the institutional framework, with participation from various social forces, both from the educational, religious, and cultural sectors, manifesting themselves as obstacles in accessing information and limiting responsible sexuality practices.

The significant loss in women’s expectations in terms of their position on the social scale (related to school dropout and low income) and in general with respect to human development is highlighted. This finding is consistent with what is reported by the Gender Affairs Observatory in Colombia [24]. In the narratives of the young people and the various study participants, there is a sense of masculine dominance, where being a man implies taking on risky behaviors, and responsible fatherhood is not a goal. Therefore, the woman is blamed and held responsible for the adolescent pregnancy. These social meanings, typical of a macho culture, have also been identified in various studies on youths’ perception, such as those published by Alvaré et al. [14] and Cabrera and Vázquez [13]. This is how the feminization of adolescent pregnancy occurs, and according to Bermúdez, tensions and inequitable processes with social gender gaps that cause inequality are revealed [10].

Regarding the scenario of violence and the analysis proposed by Galtung and Fischer [17], this research study also highlights how structural violence is reflected within the community, seen in the narratives on the state’s lack of investment in the Amaime river basin. In terms of cultural violence, there are relationships of dominance closely related to a patriarchal tradition and the lack of equity in gender roles, with a disadvantage for women in the studied territories. Gender violence triggers other types of symbolic, psychological, and economic violence, where stereotypes and social stigmas are generated that limit women’s life project options, directing them exclusively toward motherhood, and that tend to exercise control over the female body, pigeonholing them into a reproductive role [23].

### Policy recommendations and required interventions

In this research study, the young people and the other study participants demanded action and greater social, economic, and cultural opportunities (see Table 5), which can guide decision-making for local policies and in the required health interventions (see Table 6).

**Table 5:** General demands of the study participants and proposals for responses, Amaime river basin, 2019

Demands	Findings	Response program/policy
Adolescent care	The transition from childhood to adolescence requires support. In the rural context studied, drug use is perceived both in the plains and in the mountains.	Youth recreation and culture programs, protection system for children in rural areas.
Technology	There are technological barriers, as although there is a national program with government centers for Internet communication, coverage is limited. In remote rural areas, these centers are closed for long periods of time and access is not adequate for the entire population, especially for older adults and young people.	Take advantage of new information and communication technologies for education programs, improve communication networks (internet and telephone connection)
The National Learning Service (SENA)	SENA is recognized as the government institution for training in different knowledge areas, although their offering of discontinuous courses in repetitive subjects is criticized. Technical and technological programs must have coverage in the rural area.	Transfer technological knowledge to rural areas with relevant programs that address youth expectations
The political dynamics	Disappointment is expressed with respect to local politicians, as they only approach the population during election times. The municipality of El Cerrito does not give adequate responses to the social needs of rural areas, while a better response is perceived from the municipality of Palmira.	Social policies with an agenda of priorities and actions shared among the municipalities (El Cerrito and Palmira).

Source: Created by the authors

**Table 6:** Health care requirements of the study participants

Requirements	Findings	Response program/policy
Demystify beliefs and gender roles	Young people say that beliefs must be transformed to talk more freely or without taboos about relationships and sexual behavior. There is a patriarchal prejudice that promotes the belief that men are happy to have many women and get them pregnant, change partners frequently without taking responsibility for paternity, and pressure women to have sex.	Gender Equity Program, schools for parents and education for children on sexual and reproductive health based on gender equality
Marriage at an early age	Young people argue that romantic relationships at an early age shorten the time of experiencing their youth and suddenly becoming a husband or wife, with the complexities that this status involves. In the narratives, greater freedom is perceived for men in pursuing their life project, while there are more restrictions for women who assume the role of mothers as they become pregnant as adolescents, and they do not have other options or greater opportunities in their life project.	Decisions and responsible sexual behavior promotion programs aimed at strengthening young people’s life projects.
Managing contraceptives and health care centers	The limited knowledge of contraceptive methods is mentioned. Health care centers remain closed for long periods of time, with few personnel in charge of promoting health, and adults are criticized for not building adequate ties of trust. The strategy of staff members that travel to the rural area is considered insufficient, due to the monthly frequency in the El Cerrito area and the bi-monthly visits in the Palmira area.	Programs on Sexual and Reproductive Health rights, Primary Care Strategy with a renewed or comprehensive approach
Community solidarity and the violence scenario caused by the armed conflict.	The study participants feel that they live in isolation, with loss of a community bond and little communication and integration. The community was fragmented by the armed conflict, being exacerbated by the territorial division of the municipalities and the isolated geographic location. Young people do not feel that the groups involved in the armed conflict are related to early pregnancy, while adults find the issue of gender violence and its relationship with pregnancy in the armed conflict to be complex problems that need to be approached appropriately.	Promote the bond of solidarity by integration and community empowerment programs, psychosocial support for the population on issues related to overcoming violence due to the armed conflict

Source: Created by the authors

In matters of institutional responses, adolescent pregnancy must be addressed. It is possible to develop intersectoral programs to protect children and support families in providing care for young people, especially to promote an understanding of the changes in the transition from childhood to adolescence and to guide decision-making with responsible sexual behaviors and relationships, strengthening young people’s life projects. Leisure, recreation, and cultural programs are also needed to promote youth talent and the appropriate use of free time.

Faced with the issue of social networks mediated by the Internet, support from parents and teachers with these tools is essential, defining the limits that must be taken in function of the type of content, in order to give them a good use for new information and communication technologies, such as guiding their application to develop technical and technological programs with government institutions (this is the case of SENA for the Colombian rural area). Educational reinforcement is also needed to promote family communication, express feelings, and build solid emotional ties with young people.

When considering the shortcomings of the general social context that is demonstrated in this research, constructing a social policy with a shared governmental agenda between the two municipalities (Palmira and El Cerrito) is considered important, given that the Amaime river basin is unique to its isolated rural territory with a highly dispersed population. Special attention should be paid to this as they present fragility and social vulnerability due to the trajectory of the violent conflict by the armed actors that were present there. The development of social capital is an aspect recommended in other studies on the subject of adolescent pregnancy [7].

With regard to responses to health interventions, it is also important to highlight the issue of the school for parents or to develop a “Healthy Schools” strategy, incorporating projects aimed at promoting health and preventing phenomena such as pregnancy at an early age. In this socialization space, the inappropriate beliefs of groups can be demystified, addressing topics considered taboo, as found with sexual relations in rural populations. At the same time, it is important to transform the dominant relationships of patriarchy and sexism, generating more horizontal relationships between men and women through programs that promote gender equality and healthy coexistence [23].

For health care services and with a public health approach, a response based on the Primary Care Strategy in Renewed Health is a priority [25], developing programs in Health Prevention and Promotion that adjust to the characteristics of specific social contexts and their specific dynamics [26], as found in the present study. In accordance with the lines of action of the Ottawa Charter, parallel to the promotion of healthy individual behaviors, the need for population-based actions that emphasize the most vulnerable subgroups, which transcend traditional health sector interventions that focus on an individual approach, without analyzing the social context in which the phenomenon occurs [6, 27], is recognized. In rural areas, it is important that progress is made in implementing programs to promote Sexual and Reproductive Rights, in which society’s participation is expanded with more constructivist approaches.

In this sense, there are experiences in rural areas that have shown the approach to health problems and their social determinations, through education processes and social participation projects in health, with alliances between social, academic, and institutional actors committed to supporting population groups, in order to achieve the required changes [28].

A key aspect of the response to the complexity of the social process in which the phenomenon of adolescent pregnancy is immersed is to develop interventions aimed at empowering and strongly promoting community ties in order to recover social solidarity, such as the promotion of community integration and meeting programs to increase communication between neighbors and increase the positive perception about the place, the community, and diversity. In addition, it is important to generate psychosocial support for adults to overcome violence and the fragmented social fabric in the armed conflict, thus creating a favorable context in territorial management for peacebuilding [29].

### Limitations of the study and future research

Considering the limitations described in this study’s sampling, future research may expand the population framework with inclusive criteria to involve the participation of parents, grandmothers, and other caregivers of young people with experience in adolescent pregnancy. In addition, the owners of the farms and large sugarcane companies in the territory should be involved, and participation from ex-combatants from the various groups in the armed conflict should be considered. It is key to direct research questions toward this last social actor to study and be able to approach the phenomenon of adolescent pregnancy with actors of the armed conflict. It is also vital to further study the various types of violence, including structural, symbolic, and cultural violence.

## Conclusions

This research found that structural living conditions (especially economic relationships) and group ways of life (predominant patriarchal culture) are related to personal history and individual behaviors, and they become mediating social forces of the life project and youthful uncertainty regarding this. Together, these aspects are combined to influence and reproduce the phenomenon of adolescent pregnancy in rural settings, with complex social processes, such as those shown in the study.

Living conditions with strong social stigma were found due to the context of armed violence resulting from the previous political conflict, and demands for social, political, and cultural opportunities were generated. In terms of lifestyles, little communication and information on sexual education is perceived, with women being pigeonholed into a reproductive role from an early age. As for lifestyles, there are youthful behaviors infused by sociocultural traditions that affect life projects and sexual behavior. Gender relations are precarious, and there are various types of violence that limit effective peacebuilding.

Among the most representative findings are those on structural violence, present in the accounts from study participants, indicating a low institutional presence of the Colombian government, with limited public health actions and little interaction with health personnel. At the same time, cultural violence is identified in the study, which is based on a patriarchal culture that favors the role of a mother, socially assigned to women, with men taking little responsibility in fatherhood, giving rise to gender inequalities. Promoting the recognition and deconstruction of the various forms of violence would contribute to the local exercise aimed at the transition toward peacebuilding, with a healthy coexistence.

This study contributes to understanding the phenomenon of adolescent pregnancy, identifying its link to various perceived conditioning factors. Analytical elements are generated where demands are identified from the social actors involved. These findings contribute to the search for comprehensive interventions in which efforts of the entire social group are combined under a collective understanding of public health, as proposed by the Social Determination approach [16]. In this approach, an organized social response is then proposed, where the idea of isolated events is overcome and, on the contrary, health problems are recognized as phenomena immersed in complex social processes.

From the health sector, it is essential to intervene with comprehensive collective actions in the country’s post-conflict period, which is the population’s right and a governmental obligation in order to contribute to the deconstruction of armed violence and the demystification of other types of violence that are invisible (according to Galtung and Fisher’s approaches). The diversity of violence ultimately has repercussions on structural living conditions, lifestyles of specific groups, and individual behavioral styles. In turn, these deepen specific problems such as the phenomenon of adolescent pregnancy explored in this study.

By considering the perceptions of young people and the rest of the social actors on this phenomenon, the need to broaden the logic of intervention is understood, where promoting health generates local responses for the entire social group. It requires the involvement of both sectoral actions in health as well as generating trans-sectoral interventions (incorporating, for example, the educational, political, and cultural sectors as expressed by the demands of the participants in this study) and incorporating community actions.

A holistic approach, such as that of social determination applied in this study [30], can be used to create analytical elements that guide the identification of the social forces involved in a specific priority health problem in order to find options for changes required and demanded by the participants. With the actions aimed at influencing and reducing the phenomenon of adolescent pregnancy, there is a need for a joint construction aimed at achieving the required social transformations.

## Supplementary Information

**Additional file 1.**

## Data Availability

The data that support the findings of this study are available from the corresponding author on reasonable request.

## Abbreviations

FARC-EP: Fuerzas Armadas Revolucionarias de Colombia - Ejército del Pueblo [Spanish]. Revolutionary Armed Forces of Colombia-People’s Army
UUC: Autodefensas Unidas de Colombia [Spanish]. United Self-Defense Forces of Colombia
