# Supplementary material for: Perceptions of adolescent pregnancy in the rural context and the Colombian armed conflict: a qualitative approach based on social determination of health
Source: Int J Equity Health. 2021 Oct 20;20:232. doi: 10.1186/s12939-021-01568-2 (PMC8527773; doi:10.1186/s12939-021-01568-2)
Supplement: Supplementary file 1 — Additional file 1. [file 12939_2021_1568_MOESM1_ESM.docx]

_
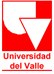
_ **GUIA PARA ENTREVISTAS SEMIESTRUCTURADAS**

**LAS PERCEPCIONES SOBRE EL EMBARAZO ADOLESCENTE EN EL CONTEXTO DEL PROCESO DE PAZ EN COLOMBIA: UN ABORDAJE DESDE LA DETERMINACIÓN SOCIAL.**

**Entrevistas Semiestructuradas**

1. **Objetivo general:**

Describir las percepciones de los jóvenes y de otros actores sociales de la cuenca del rio Amaime sobre las fuerzas sociales y dimensiones de determinación social implicadas en el embarazo en adolescente en escenarios de violencia y construcción de paz.

1. **Objetivo de las entrevistas:**

Las entrevistas semiestructuradas tienen como objetivo conocer la percepción de los actores (jóvenes, líderes, personal de salud) en la cuenca del rio Amaime sobre el embarazo en adolescente en el contexto de construcción de paz en Colombia para ser abordado desde el enfoque de la determinación social. Se busca describir el fenómeno desde herramientas de la investigación cualitativa buscando la participación de la comunidad desde la experiencia vivida del fenómeno, como un ejercicio de comprensión para la construcción y aprehensión del conocimiento.

Los tópicos de las entrevistas son los mismos, algunas preguntas tienen una variación de acuerdo al actor que se va a entrevistar.

**Guía entrevista semi estructurada** para actores juveniles

1. Tópico: Percepción sobre las Condiciones de Vida.
2. Tópico: Percepción sobre las fuerzas sociales.
3. Tópico: Percepción del Embarazo en Adolescente.
4. Tópico: Percepción sobre la Construcción de paz.

**Percepción sobre las condiciones de vida**

-¿Cuáles son las condiciones en general (contexto económico, cultural, político) de los jóvenes que habitan el territorio de la cuenca del Amaine y como se relacionan esas condiciones de vida de los jóvenes con los embarazos a edad temprana?

-¿Qué significado le otorga a las condiciones de vida, en relación o efecto positivo o negativo, con una experiencia de embarazo adolescente?

-¿Cómo el modo de vivir, en cuanto a aspectos colectivos específicos o características propias de los grupos de jóvenes de la Cuenca del Amaine, puede relacionarse y tener significado para que se dé un embarazo en adolescente?

- -¿Cómo el estilo de vida, en cuanto a comportamientos, hábitos sexuales y reproductivos, puede relacionarse y tener significado para que se dé un embarazo en adolescente?
- ¿Cómo la identidad o consciencia de género en cuanto a masculinidad/feminidad, se relacionan con el embarazo adolescente?

**Percepción sobre las Fuerzas sociales:**

-¿Cuál es el papel de la familia frente al riesgo de los jóvenes a embarazarse en la adolescencia?

-¿Qué papel juega la familia en la experiencia de los hijos en el embarazo en adolescentes?

-¿Qué papel desempeña la comunidad de la Cuenca del Amaine o que actores sociales identifica en términos de la prevención del embarazo en los adolescentes?

-¿Cómo la experiencia de vivir en la cuenca del Amaime y no en otro lugar determina la experiencia del embarazo a edad temprana?

**Percepción del embarazo en adolescente**

-¿Cuáles consideran sean las causas que originan el embarazo en adolescente en la cuenca del rio Amaime?

-¿Cómo se percibe a un joven cuando se embaraza a edad temprana en la cuenca del rio Amaime de acuerdo a la experiencia? (aspectos positivos-ventajas y/o negativos-desventajas o problemas).

-¿Que creencias se tienen en comunidad sobre el embarazo en adolescente?

-¿Cómo se vive el embarazo de una adolescente desde su experiencia?

**Percepción sobre la Construcción de paz**

-¿Qué tipos de violencia generan un embarazo en adolescencia en el territorio?

-¿De qué manera los procesos de violencia en un territorio se relacionan con la experiencia del embarazo adolescente?

-¿Cómo percibe el proceso de paz con Las Farc Ep?

-¿Cómo se pueden generar cambios de percepción en el caso del embarazo en adolescente al desmovilizarse las Farc-Ep y avanzar en un proceso de construcción de paz?

_
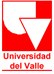
_ **LAS PERCEPCIONES SOBRE EL EMBARAZO ADOLESCENTE EN EL CONTEXTO DEL PROCESO DE PAZ EN COLOMBIA: UN ABORDAJE DESDE LA DETERMINACIÓN SOCIAL.**

**Entrevista semi estrucurada a Personal de Salud**

**Percepción sobre las condiciones de vida**

-¿Cuáles son las condiciones en general (contexto económico, cultural, político) de los jóvenes que habitan el territorio de la cuenca del Amaine y como se relacionan esas condiciones de vida de los jóvenes con los embarazos a edad temprana?

-¿Qué significado le otorga a las condiciones de vida, en relación o efecto positivo o negativo, con una experiencia de embarazo adolescente?

-¿Cómo el modo de vivir, en cuanto a aspectos colectivos específicos o características propias de los grupos de jóvenes de la Cuenca del Amaine, puede relacionarse y tener significado para que se dé un embarazo en adolescente?

-¿Cómo el estilo de vida, en cuanto a comportamientos, hábitos sexuales y reproductivos, puede relacionarse y tener significado para que se dé un embarazo en adolescente?

- ¿Cómo la identidad o consciencia de género en cuanto a masculinidad/feminidad, se relacionan con el embarazo adolescente?

**Percepción Sobre las Fuerzas sociales:**

¿Cómo el lugar cuenca del Amaime se vuelve en un escenario propicio para que se den tipos de relación que inciden en el embarazo en adolescente?

¿Qué tipo de programas en salud sexual y reproductiva se realizan en estas comunidades que involucren los derechos sexuales?

¿Se tiene planteado y/o ya se ha implementado una estrategia de intervención para mitigar el embarazo en adolescente de manera intersectorial en la cuenca del Amaime?

**Percepción del embarazo en adolescente:**

¿Cuáles son las causas o factores que aumentan el embarazo en adolescente en los jóvenes de la cuenca del Amaime?

**Percepción sobre la Construcción de paz**

¿Cómo considera usted que la construcción de la paz el desarme de las Farc-Ep puede el problema del embarazo en adolescente?

¿Cómo generar capacidad de incidencia a nivel de los jóvenes en los temas de promoción de la salud y prevención del embarazo en adolescente?

¿Qué capacidades reconoce usted en la comunidad para crear o reproducir valores culturales que protejan el riesgo del embarazo en adolescente?

_
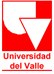
_**LAS PERCEPCIONES SOBRE EL EMBARAZO ADOLESCENTE EN EL CONTEXTO DEL PROCESO DE PAZ EN COLOMBIA: UN ABORDAJE DESDE LA DETERMINACIÓN SOCIAL.**

**Entrevista semi estructurada a actores, líderes habitantes de la cuenca del rio Amaime.**

1. **Percepción sobre las condiciones de vida:**

-¿Cuáles son las condiciones en general (contexto económico, cultural, político) de los jóvenes que habitan el territorio de la cuenca del Amaine y como se relacionan esas condiciones de vida de los jóvenes con los embarazos a edad temprana?

-¿Qué significado le otorga a las condiciones de vida, en relación o efecto positivo o negativo, con una experiencia de embarazo adolescente?

-¿Cómo el modo de vivir, en cuanto a aspectos colectivos específicos o características propias de los grupos de jóvenes de la Cuenca del Amaine, puede relacionarse y tener significado para que se dé un embarazo en adolescente?

-¿Cómo el estilo de vida, en cuanto a comportamientos, hábitos sexuales y reproductivos, puede relacionarse y tener significado para que se dé un embarazo en adolescente?

- ¿Cómo la identidad o consciencia de género en cuanto a masculinidad/feminidad, se relacionan con el embarazo adolescente?

1. **Percepción sobre las Fuerzas sociales:**

-¿Cuál es el papel de la familia frente al riesgo de los jóvenes a embarazarse en la adolescencia?

-¿Qué papel juega la familia en la experiencia de los hijos en el embarazo en adolescente

-¿Qué papel desempeña la comunidad del territorio de la cuenca del Amaine en términos de la prevención del embarazo en los adolescentes?

-¿Cómo la experiencia de vivir en la cuenca del Amaime y no en otro lugar determina la experiencia del embarazo a edad temprana?

-¿Cuál es el papel de los lideres, los grupos y las organizaciones ante el tema del embarazo en adolescente?

-¿Cuál es el rol del sistema de salud frente al embarazo en adolescente en la cuenca del Amaime?

1. **Percepción del Embarazo en adolescente:**

-¿Cuáles consideran sean las causas que originan el embarazo en adolescente en la cuenca del rio Amaime?

-¿Cómo se percibe a un joven cuando se embaraza a edad temprana en la cuenca del rio Amaime de acuerdo a la experiencia? (aspectos positivos-ventajas y/o negativos-desventajas o problemas).

-¿Que creencias se tienen en comunidad sobre el embarazo en adolescente?

¿Qué percepción se tiene ante el embarazo en adolescente en la cuenca del Amaime?

4**. Percepción sobre la Construcción de paz**

1. ¿Qué relación puede tener el embarazo en adolescente con la construcción de paz ante el desarme de las FARC-EP?

2. ¿Qué relación puede tener las violencias con el embarazo en adolescente?

3. ¿Cómo evitar que los jóvenes se embaracen a edad temprana?

4. ¿Cómo vincular programas educativos en la comunidad para que los jóvenes prevengan el embarazo en adolescente?

_
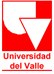
_ **GUÍA PARA EL GRUPO FOCAL**

**LAS PERCEPCIONES SOBRE EL EMBARAZO ADOLESCENTE EN EL CONTEXTO DEL PROCESO DE PAZ EN COLOMBIA: UN ABORDAJE DESDE LA DETERMINACIÓN SOCIAL.**

1. **Objetivo general de la investigación:**

Describir las percepciones de los jóvenes y de otros actores sociales de la cuenca del rio Amaime sobre las fuerzas sociales y dimensiones de determinación social implicadas en el embarazo en adolescente en escenarios de violencia y construcción de paz.

1. **Objetivo del grupo focal:**

El grupo focal tiene como objetivo conocer la percepción de los actores de la cuenca del Amaime sobre el embarazo en adolescente en el contexto de construcción de paz en Colombia desde el enfoque de la determinación social. Se busca analizar el fenómeno desde herramientas de la investigación cualitativa buscando la participación de la comunidad desde la experiencia vivida del fenómeno para la construcción y aprehensión del conocimiento.

1. Moderador (Nombre y apellidos):____________________________________
2. Observador (Nombre y apellidos):___________________________________

Listado de participantes:

| Número | Nombre y apellido | comunidad | Teléfono | Firma |
| --- | --- | --- | --- | --- |
| 1 |  |  |  |  |
| 2 |  |  |  |  |
| 3 |  |  |  |  |
| 4 |  |  |  |  |
| 5 |  |  |  |  |
| 6 |  |  |  |  |
| 7 |  |  |  |  |
| 8 |  |  |  |  |
| 9 |  |  |  |  |
| 10 |  |  |  |  |
| 11 |  |  |  |  |
| 12 |  |  |  |  |

**Tópico para el grupo focal:**

Tópico 1: Percepción sobre las Condiciones de Vida.

Tópico 2 Espacios de Reproducción Social.

Tópico 3: Percepción del Embarazo en Adolescente.

Tópico 4: Construcción de paz.

**Percepción sobre las condiciones de vida**

-¿Cuáles son las condiciones en general (contexto económico, cultural, político) de los jóvenes que habitan el territorio de la cuenca del Amaine y como se relacionan esas condiciones de vida de los jóvenes con los embarazos a edad temprana?

-¿Qué significado le otorga a las condiciones de vida, en relación o efecto positivo o negativo, con una experiencia de embarazo adolescente?

-¿Cómo el modo de vivir, en cuanto a aspectos colectivos específicos o características propias de los grupos de jóvenes de la Cuenca del Amaine, puede relacionarse y tener significado para que se dé un embarazo en adolescente?

-¿Cómo el estilo de vida, en cuanto a comportamientos, hábitos sexuales y reproductivos, puede relacionarse y tener significado para que se dé un embarazo en adolescente?

- ¿Cómo la identidad o consciencia de género en cuanto a masculinidad/feminidad, se relacionan con el embarazo adolescente?

**Percepción sobre las Fuerzas sociales:**

-¿Cuál es el papel de la familia frente al riesgo de los jóvenes a embarazarse en la adolescencia?

-¿Qué papel juega la familia en la experiencia de los hijos en el embarazo en adolescente?

-¿Qué papel desempeña la comunidad del territorio de la cuenca del Amaine o que actores sociales identifica en términos de la prevención del embarazo en los adolescentes?

-¿Cómo la experiencia de vivir en la cuenca del Amaime y no en otro lugar determina la experiencia del embarazo a edad temprana?

**Percepción del embarazo en adolescente**

-¿Cuáles consideran sean las causas que originan el embarazo en adolescente en la cuenca del rio Amaime?

-¿Cómo se percibe a un joven cuando se embaraza a edad temprana en la cuenca del rio Amaime de acuerdo a la experiencia? (aspectos positivos-ventajas y/o negativos-desventajas o problemas).

-¿Que creencias se tienen en comunidad sobre el embarazo en adolescente?

-¿Cómo se vive el embarazo de una adolescente desde su experiencia?

**Percepción sobre la Construcción de paz**

-¿Qué tipos de violencia generan un embarazo en adolescencia en el territorio?

-¿De qué manera los procesos de violencia en un territorio se relacionan con la experiencia del embarazo adolescente?

-¿Cómo percibe el proceso de paz con Las Farc Ep?

-¿Cómo se pueden generar cambios de percepción en el caso del embarazo en adolescente al desmovilizarse las Farc-Ep y avanzar en un proceso de construcción de paz?
